# Can Improved Biosecurity Measures Reduce the Presence of the Most Common ESBL-Producing *Enterobacteriaceae*? A Study from Greek Pig Farms

**DOI:** 10.3390/life15101629

**Published:** 2025-10-19

**Authors:** Nikolaos Tsekouras, Spyridon Antoniadis, Zoi Athanasakopoulou, Dimitris C. Chatzopoulos, Dimitrios Kantas, Vassiliki Spyrou, Georgios Christodoulopoulos, Charalambos Billinis, Vasileios G. Papatsiros

**Affiliations:** 1 Clinic of Medicine, Faculty of Veterinary Science, University of Thessaly, 43100 Karditsa, Greece; nitsekou@vet.uth.gr; 2 Department of Biology, Division of Animal and Human Physiology, National and Kapodistrian University of Athens, 15784 Athens, Greece; santonia@biol.uoa.gr; 3 Department of Microbiology and Parasitology, Faculty of Veterinary Science, University of Thessaly, 43100 Karditsa, Greece; zathanas@uth.gr (Z.A.); billinis@uth.gr (C.B.); 4 Laboratory of One Health, Infectious Diseases and Zoonoses, Department of Public and One Health, University of Thessaly, 43100 Karditsa, Greece; dchatzopoulos@uth.gr; 5 Faculty of Animal Science, University of Thessaly, 41110 Larissa, Greece; dkantas@uth.gr (D.K.); vasilikispyrou@uth.gr (V.S.); 6 Department of Animal Science, Agricultural University of Athens, 11855 Athens, Greece; gc@aua.gr

**Keywords:** antimicrobial resistance, ESBL, biosecurity, swine, Greece

## Abstract

This study investigates the relationship between biosecurity implementation and the prevalence of ESBL-producing bacteria in Greek swine farms, revealing an alarming 82% prevalence rate, which is significantly higher than in other European nations. Our findings indicate that comprehensive biosecurity measures are more effective than focusing on priority controls alone. Notably, there was a lack of significant associations between farm size and individual biosecurity parameters, underscoring the importance of cumulative improvements across multiple measures. Moreover, we identified a critical threshold for biosecurity implementation: farms scoring less than a critical level were consistently vulnerable to ESBL contamination. *Escherichia coli* emerged as the dominant species among ESBL isolates, reflecting patterns seen globally. This suggests a need for targeted biosecurity strategies, as mixed species occurrences imply shared resistance pressures. Our results advocate for a paradigm shift in biosecurity practices, emphasizing holistic improvements across all measures rather than isolating specific controls. With current implementation levels averaging only 35% of recommended biosecurity practices, our findings highlight the urgent need for comprehensive interventions to mitigate antimicrobial resistance in the Greek swine industry.

## 1. Introduction

Antimicrobials, the 20th century’s “wonder drugs” as they were called by health professionals, even in official documents in the 1950s’ [1], after less than a century of use have proven to be ineffective in many cases. While widespread and irrational use led to increased cases of antimicrobial resistance, the discovery of new antimicrobials reduced dramatically [2]. According to recent estimations, more than the half of 11,000 tons of antimicrobials sold in the United States for use in livestock belong to the “medically important” category [3].

Except increased morbidity, mortality and treatment cost for livestock, the bacteria *Enterobacteriaceae*, which produce extended-spectrum β-lactamases (ESBLs), are also a significant issue for human health [4]. The World Health Organization (WHO) characterized ESBLs as critical priority pathogens [5]. Many studies in the past, including from Greece, demonstrated a strong presence of ESBL producers in livestock and especially in pigs [6,7,8]. Transmission of ESBL-producing *Enterobacteriaceae* such as *Escherichia coli* (*E. coli*) occurs via many possible routes, such as consumption of inadequately thermally processed animal products or from live animals to humans and vice versa [9,10]. Especially, pig farms’ workers and the staff of slaughterhouses are exposed to the risk of possible intestinal colonization with antimicrobial-resistant (AMR) pathogenic bacteria. Pork meat could also be cross-contaminated by ESBL-producing bacteria by slaughterhouse personnel [11].

The term biosecurity includes all the practices adopted to prevent the entrance and spread of pathogens within a farm or group of animals. In particular, external biosecurity aims to prevent the entering of pathogens into a herd, and internal biosecurity measures reduce dissemination within a herd [12,13]. Over recent years, outbreaks of difficult to control diseases such as African Swine Fever (ASF), Porcine Reproductive and Respiratory Syndrome Virus (PRRSV) and porcine epidemic diarrhea highlighted the strong association between pig farm sustainability, pig health and biosecurity [14,15,16]. Previous studies have also revealed that high biosecurity levels can positively influence farm productivity and the economic performance of a farm [17]. Consequently, low biosecurity is associated with increased rates of clinical disease and the need for frequent treatment in farms [18]. In other livestock sectors, such as poultry, biosecurity is considered a valuable tool which promotes prudent use of antimicrobials, animal health and welfare and productivity [19]. Implementation of biosecurity and herd management, reduce the risk of infectious diseases, the need for antimicrobial treatments and the development of AMR phenomena [20].

Antimicrobial use (AMU) has led to the development of AMR bacteria that complicate treatment of infectious diseases in animals and humans. The majority of veterinary AMU occurs in pigs, which is known to contribute to the development of AMR. High AMU and the threat of AMR highlighted the need for reduced AMU in pig production [21]. European Union (EU) countries with a high AMU also rank high in their resistance levels [22]. In this context, ESBL *Enterobacteriaceae*-positive European pig farms are reported to include up to 46% of Dutch farms, 61% of German farms and 82% of Greek farms tested [8,23,24]. The level of biosecurity of a herd was associated with the amount of AMU [25]. Farm-specific preventive strategies can contribute to lowering the risk of animal disease and hence the need for AMU [26]. The improvement of biosecurity measures is an important approach to prevent the entry and spread of pathogens in a herd and thus may reduce the necessity of AMU [12,18,21].

The influence which varying biosecurity levels, may have on ESBL bacteria prevalence could aid by reducing their spread within pig populations. Comprehension of this relationship is crucial to developing and adopting strategies that could reduce antimicrobial use, leading to reduced risk of AMR pathogens through the “One Health” approach. This study aims to investigate, for first time in Greek swine farms, the possible association between biosecurity practices and the presence of ESBL-producing *Enterobacteriaceae*. Furthermore, an additional goal was to investigate how biosecurity measures, which are considered to be of secondary importance, can contribute to the reduction of ESBL-producing *Enterobacteriaceae*.

## 2. Materials and Methods

### 2.1. Study Design and Farm Selection

This cross-sectional study included 34 pig farms, representing approximately 24% of Greek swine production capacity (14,300 sows total). Farms were distributed across northern (*n* = 4), central (*n* = 13), western (*n* = 10) and southern (*n* = 7) Greece. The geographical distribution of the farms was according to pig population density across Greece. Participating farms included small-scale operations (*n* = 8 farms, <200 sows, minimum 50 sows, range: 50–180 sows), medium-scale commercial facilities (*n* = 23 farms, 200–800 sows, range: 200–750 sows), and large industrial operations (*n* = 3, >800 sows, range: 1000–2100 sows). This distribution reflects the predominance of medium-scale operations typical of modern Greek swine production systems, while retaining sufficient representation of smaller family operations and large commercial facilities to enable robust assessment across the full industry spectrum. Different biosecurity measures were applied on each farm and, as a result, biosecurity levels varied between them. The main criteria for the farms to participate in this study were as follows: (i) All farms included in this study operated exclusively as farrow-to-finish systems. (ii) Their diets consisted of home-formulated rations based on mixed corn/barley/wheat–soybean meal, balanced for essential amino acids, minerals and vitamins in accordance with the Nutrient Requirements of Swine [27]. (iii) In addition, farm selection was based on standardized vaccination protocols against common pathogens. These included (a) sows: Aujeszky’s disease virus, Porcine parvovirus, Atrophic rhinitis, Erysipelas, Porcine Reproductive and Respiratory Virus, *Escherichia coli* and *Clostridium* spp.; and (b) weaners: porcine circovirus type 2 and *Mycoplasma hyopneumoniae*. Not all pig farms in each geographical region participated in the study and participation was primarily based on random selection of the farms, with the farmers’ willingness to participate serving as a secondary criterion. Biosecurity implementation was evaluated, by a quostinnaire providing at Supplementary File S1, using a scoring system developed for this study, with 35 parameters selected based on Silva et al. [28]. The system employed a weighted approach differentiating between 14 priority parameters (scored 0, 5, or 10 points each; maximum 140 points) and 21 supplementary parameters (scored 0 or 5 points each; maximum 105 points), for a total possible of 245 points. This three-tier scoring for priority measures (0 = absent, 5 = partial implementation, 10 = full implementation) was designed to reflect the discrete nature of how biosecurity measures exist in field conditions while enhancing inter-rater reliability.

Biosecurity level assessments were conducted by experienced veterinarians in our team during the initial farm visits, incorporating the previous 5 years of biosecurity history as reported by farmers. The specific parameters evaluated are detailed in Table 1 and Table 2.

### 2.2. Sample Collection and ESBL Detection

#### 2.2.1. Sample Collection

A total of 214 fecal samples were collected from 73 suckling and 141 weaning piglets across the 34 farms between December 2019 and April 2021. Samples were obtained directly from the rectum using swabs with Amies transport medium and processed within 24 h.

#### 2.2.2. ESBL Detection and Characterization

ESBL-producing *Enterobacteriaceae* detection followed the methodology described in Tsekouras et al. [8]. Briefly, fecal swabs were directly streaked on ESBL-selective media (CHROMID^®^ ESBL, BioMérieux, Marcy l’Etoile, France) and incubated aerobically at 37 °C for 24–28 h. Bacterial species identification was carried out using the automated Vitek-2 system (BioMérieux, Marcy l’Etoile, France), according to the manufacturer’s instructions.

Antimicrobial susceptibility testing of all the obtained strains was performed by the Vitek-2 system. The AST-GN96 card was used to determine the Minimum Inhibitory Concentration (MIC) of the following antimicrobial agents: ampicillin, amoxicillin/clavulanic acid, ticarcillin/clavulanic acid, cefalexin, cefalotin, cefoperazone, ceftiofur, cefquinome, imipenem, gentamicin, neomycin, flumequine, enrofloxacin, marbofloxacin, tetracycline, florfenicol, polymyxin B and trimethoprim/sulfamethoxazole. Results were automatically interpreted using Vitek-2 software (BioMérieux, system version 8.02).

Phenotypic confirmation of ESBL production was performed using the double disk synergy test (DDST) or combination disk test (CDT) according to EUCAST guidelines [8].

Isolation of *Salmonella* spp. was conducted according to ISO 6579-1:2017 [29]. Initially, swabs were agitated and squeezed into sterilized tubes containing 9 mL Buffered Peptone Water (BPW). Subsequently, Modified Semisolid Rappaport Vassiliadis (MSRV) agar, Xylose Lysine Deoxychocolate (XLD) agar and Salmonella Shigella (SS) agar was used as selective media under the recommended conditions. All presumptive *Salmonella* colonies were identified by species using the Vitek-2 system.

All the isolates and their antimicrobial susceptibility are presented at Supplementary File S2.

### 2.3. Statistical Analysis

#### 2.3.1. Data Transformation

Due to pronounced right skewness of sow capacity data (skewness = 2.46), natural log transformation was applied:

C’ = ln(C)

where C represents raw sow capacity and C’ the transformed variable. This transformation normalized the distribution, reduced the influence of outliers and stabilized variance across scales.

#### 2.3.2. Normality Testing

Distribution normality was assessed using the Shapiro–Wilk test. Variables violating normality assumptions (*p* < 0.05) were analyzed using non-parametric methods.

#### 2.3.3. Association Analyses

Group comparisons between ESBL-positive and ESBL-negative farms were performed using Mann–Whitney U tests for continuous biosecurity scores, with effect sizes calculated using the formula r = Z/√((*n*) to assess the magnitude of observed differences. Correlation analyses employed Spearman rank correlations to assess relationships between continuous variables that violated normality assumptions, while point-biserial correlations were used to evaluate associations between the binary outcome of ESBL presence and continuous biosecurity scores. Regression modeling followed a hierarchical approach to examine different aspects of the biosecurity–ESBL relationship. Linear regression models examined the influence of farm size (log-transformed sow capacity) on biosecurity implementation levels, with model fit assessed using adjusted R^2^ values. Logistic regression models evaluated the predictive relationship between biosecurity scores and ESBL detection. The logistic regression implementation uses maximum likelihood estimation, with *p*-values calculated via Wald statistics. Results are expressed as odds ratios obtained by exponentiating the coefficients (OR = exp(β)), with 95% confidence intervals calculated as exp(β ± 1.96×SE). Model performance for logistic regression was assessed using Nagelkerke’s R^2^, which provides a measure of the proportion of variance explained analogous to R^2^ in linear regression.

To investigate associations between farm size and individual biosecurity parameters, ordinal logistic regression was employed. This approach was necessary because individual biosecurity parameters are scored as ordered categories (0/5/10 for priority measures; 0/5 for supplementary measures) rather than continuous values. Given the exploratory nature of testing 35 separate parameters, False Discovery Rate (FDR) correction using the Benjamini–Hochberg procedure was applied to control for Type I errors while maintaining statistical power. This method controls the expected proportion of false discoveries among rejected hypotheses, providing a less conservative alternative to traditional family-wise error rate corrections.

All analyses were performed using MATLAB R2025a with significance set at α = 0.05. Model performance was assessed using Nagelkerke’s R^2^ for logistic models and adjusted R^2^ for linear models.

## 3. Results

### 3.1. Farm Operational Characteristics

#### 3.1.1. Farm Size Distribution

**Figure 1:**
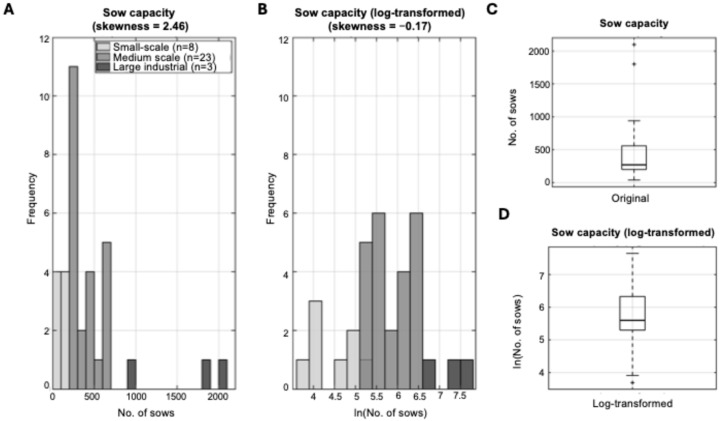
Distribution of sow capacity across 34 pig farms located in different geographical regions of Greece. (**A**) Raw sow capacity data showing strong right skewness. Each histogram bin is color-coded according to the production scale of farms within that capacity range. This color scheme illustrates how farm size categories are distributed across the sow capacity spectrum and is for visualization purposes only; all statistical analyses used continuous sow capacity values. (**B**) Log-transformed sow capacity data demonstrating its effectiveness in achieving near-perfect symmetry. The transformation successfully normalized the heavily skewed distribution, reduced the disproportionate influence of large-scale outliers, stabilized variance across the full range of production scales, and created a distribution suitable for parametric statistical analyses. (**C**) Box plot of raw sow capacity data illustrating the presence of extreme outliers (black circles). (**D**) Box plot of log-transformed data showing reduced outlier influence and more balanced distribution around the median.

Farm sow unit capacity demonstrated considerable heterogeneity across the 34 participating operations, ranging from 40 to 2100 sows (median: 270, IQR: 200–560; Figure 1C). This reflected an approximately 52-fold difference in production scale. Examination of the raw capacity data revealed that most farms clustered at small to medium scales, while a small number of large facilities extended the upper range, producing a strongly right-skewed distribution (skewness = 2.46; Figure 1A).

The log transformation successfully normalized the heavily skewed distribution (Figure 1B), reduced the disproportionate influence of large-scale outliers, and yielded a distribution suitable for parametric statistical analyses (median: 5.6, IQR: 5.3–6.3; Figure 1D). This transformation was essential for subsequent correlation and regression analyses.

#### 3.1.2. Biosecurity Implementation Levels

**Figure 2:**
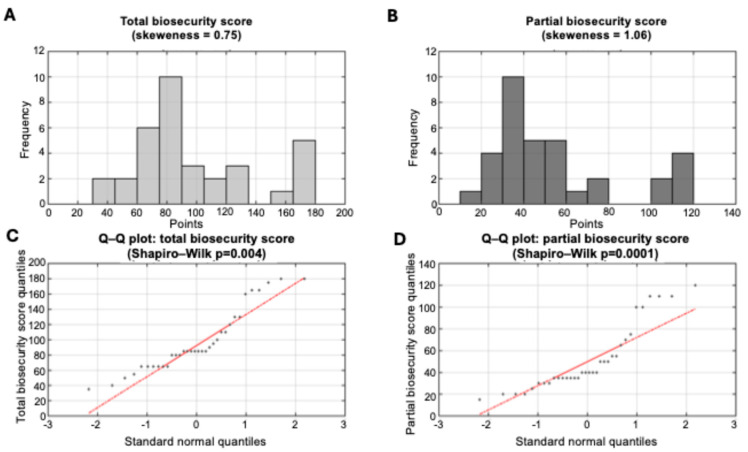
Biosecurity score distributions and normality assessment. (**A**) Histogram showing the distribution of total biosecurity scores across the 34 participating farms. (**B**) Histogram showing the distribution of priority biosecurity scores (14 most important parameters). (**C**) Quantile–quantile plot comparing total biosecurity score quantiles against theoretical normal distribution quantiles; deviation from the diagonal reference line (in red) indicates departure from normality. Grey points represent observed data quantiles, while the black line represents the expected pattern under normal distribution assumptions. (**D**) Quantile–quantile plot for priority biosecurity scores showing comparison with theoretical normal distribution. The curved patterns observed in both Q–Q plots confirm significant departure from normality, as indicated by Shapiro–Wilk test results (*p* < 0.01 for both distributions).

Total biosecurity scores demonstrated substantial variation across participating farms, ranging from 35 to 180 points (median: 85, IQR: 65–120) out of a maximum possible 245 points (Figure 2A). This represented an approximately 5-fold difference in biosecurity implementation quality, with scores spanning from 14% to 73% of the maximum possible score (median: 35%). Approximately 76% of farms scored below 50% of the maximum possible points, indicating that while some achieved relatively systematic biosecurity implementation, the majority demonstrated moderate-to-substantial deficiencies in recommended practices.

An interquartile range of 55 points (65–120) encompasses farms with markedly different biosecurity philosophies and implementation capabilities. Farms in the lower quartile represent operations with fundamental biosecurity gaps across multiple domains, while those in the upper quartile demonstrate more systematic approaches to pathogen prevention, though still falling short of comprehensive implementation

The priority biosecurity score, consisting of the 14 most important recommended biosecurity measures ranged from 15 to 120 points (median: 40, IQR: 35–65) out of a maximum possible 140 points, representing an 8-fold difference in core biosecurity implementation (Figure 2B). This distribution demonstrated that participating farms achieved substantially lower performance on priority measures compared to their overall biosecurity implementation, with a priority biosecurity score median of 29% compared to 35% for total scores.

Notably, the 8-fold variation in priority biosecurity scores exceeded the 5-fold variation observed in total scores, indicating greater heterogeneity in core measure implementation compared to supplementary biosecurity practices. Given that these 14 parameters demonstrated greater variability, although representing 57% of total possible biosecurity points (140/245), core biosecurity implementation appears more resource-dependent or technically challenging than secondary measures.

Assessment of biosecurity score distributions revealed moderate positive skewness for total biosecurity scores (skewness = 0.75) and more pronounced skewness for priority biosecurity scores (skewness = 1.06). Despite moderate skewness, the Shapiro–Wilk test rejected normality for total scores (*p* = 0.004; Figure 2C) and showed even stronger departures for priority scores (*p* = 0.0001; Figure 2D).

#### 3.1.3. Impact of Farm Size on Biosecurity Implementation Practices

Given the substantial heterogeneity in both farm size and biosecurity implementation across the 34 participating operations, we investigated whether farm size influenced biosecurity practices. Spearman rank correlation analysis revealed strong positive associations between sow capacity and biosecurity implementation. Sow capacity demonstrated a strong correlation with both total (ρ = 0.687, *p* < 0.001) and priority biosecurity scores (ρ = 0.628, *p* < 0.001), indicating that farms with greater sow capacity consistently achieved higher biosecurity standards.

**Figure 3:**
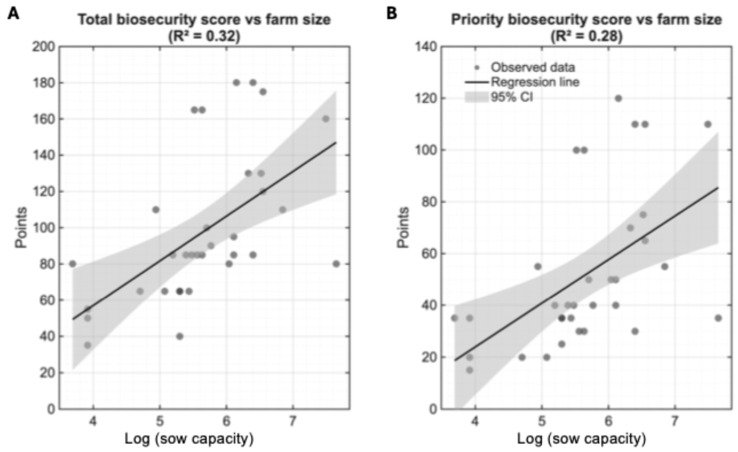
Relationship between farm size (log-transformed sow capacity) and biosecurity implementation levels. (**A**) Total biosecurity scores (maximum possible: 245 points) demonstrated a positive correlation with sow unit capacity (Spearman ρ = 0.687, *p* < 0.001), with linear regression explaining 32% of the variance. (**B**) Priority biosecurity scores, representing the 14 most critical biosecurity measures (maximum possible: 140 points), showed a similar but slightly weaker association with farm size (Spearman ρ = 0.628, *p* < 0.001) with 28% of variance explained. Black lines represent fitted linear regression models with 95% confidence intervals shown in gray shading, indicating the uncertainty in the predicted relationship. Wider intervals at the extremes of farm size reflect fewer observations at farms with very small and very large sow capacity. Each point represents an individual farm.

Linear regression analysis using log-transformed sow capacity confirmed these relationships as statistically significant predictors of biosecurity implementation. Sow capacity explained 32% of the variance in total biosecurity scores (R^2^ = 0.32, *p* < 0.001; Figure 3A) and 28% of the variance in priority biosecurity scores (R^2^ = 0.28, *p* < 0.001; Figure 3B). The 95% confidence intervals widen at both extremes of the log-transformed scale, reflecting greater uncertainty at the smallest and largest farms (in terms of sow capacity), where data are sparser. Despite this uncertainty, the consistently positive slopes and non-overlapping confidence bands across most of the ranges confirm robust associations.

#### 3.1.4. Farm Size Associations with Individual Biosecurity Parameters

To identify specific biosecurity measures influenced by production scale, we examined associations between log-transformed sow capacity and scores for all 34 biosecurity parameters using ordinal logistic regression. Given the exploratory nature of this analysis, we applied FDR correction using the Benjamini–Hochberg procedure to control for multiple comparisons.

**Figure 4:**
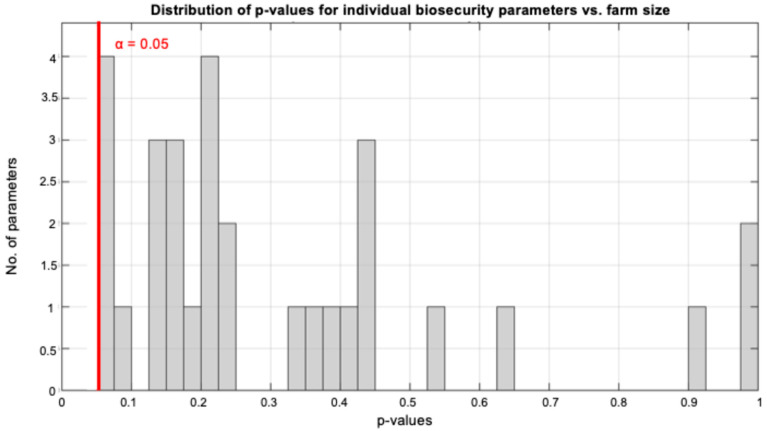
Distribution of *p*-values from ordinal logistic regression analysis testing associations between farm size and individual biosecurity parameters. Despite the strong overall correlation between farm size and total biosecurity scores (ρ = 0.687, *p* < 0.001; see Figure 5A), none of the 35 individual biosecurity parameters achieved statistical significance (all *p* > 0.05, indicated by red solid line). The smallest observed *p*-value was 0.052, and after FDR correction for multiple testing, all adjusted *p*-values exceeded 0.45. This finding reveals that superior biosecurity in farms with greater sow capacity results from distributed marginal improvements across multiple parameters rather than excellence in specific measures.

Remarkably, despite the strong overall correlation between farm size and total biosecurity scores (ρ = 0.687, *p* < 0.001; Figure 3A), no individual biosecurity parameter showed a statistically significant association with farm size (Figure 4), even before multiple testing correction. The smallest *p*-value observed was 0.052 for parameter P5, with all *p*-values exceeding the conventional significance threshold of 0.05. After FDR correction, all adjusted *p*-values exceeded 0.45, confirming the absence of significant individual parameter associations.

This paradoxical pattern—a strong aggregate relationship without significant individual component associations—indicates that farms with greater sow capacity achieve superior biosecurity through cumulative marginal improvements distributed across the entire spectrum of biosecurity measures rather than through excellence in specific parameters. Each individual improvement may be too small to achieve statistical significance, yet their combined effect produces the strong overall association observed.

### 3.2. ESBL Prevalence and Species Distribution

**Figure 5:**
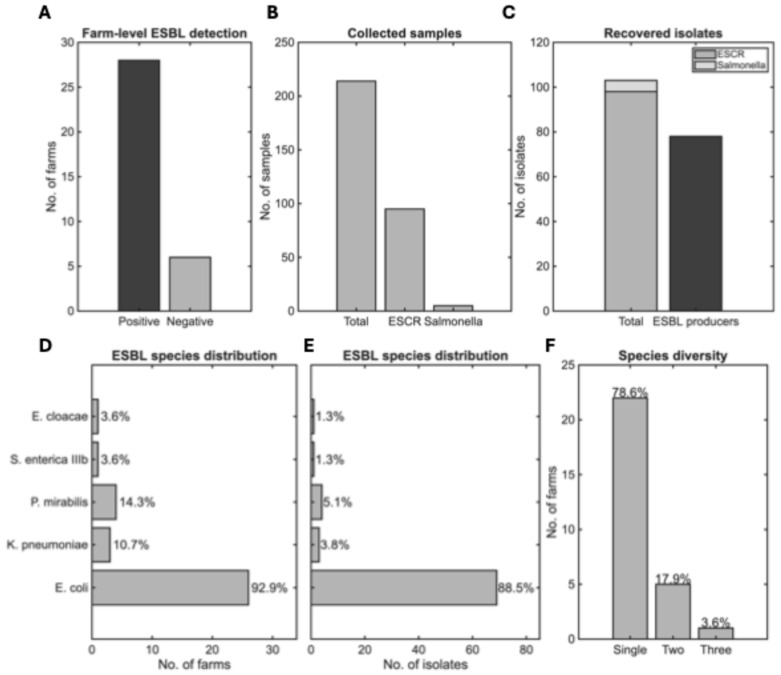
Prevalence, species distribution, and co-occurrence of ESBL bacteria across the 34 participating operations. (**A**) Number of farms with at least one ESBL-producing isolate detected (Positive, 82.35%) compared with farms where no ESBL-producing isolates were detected (Negative, 17.65%). (**B**) Total collected samples, including ESCR and *Salmonella* samples. (**C**) ESCR, *Salmonella* and ESBL-producing isolates. (**D**) Distribution of ESBL species per farm, with percentages. (**E**) Distribution of ESBL-producing isolates per species. The *y*-axis labels are the same as in (**D**). (**F**) ESBL species diversity per farm. Single-species infections predominated (*n* = 22), with *E. coli* as the most common isolate.

ESBL-producing *Enterobacteriaceae* were detected in 28 out of 34 farms (82%), indicating substantial prevalence within the study population (Figure 5A). From these ESBL-positive farms, a total of 98 extended-spectrum cephalosporin-resistant (ESCR) strains were recovered by selective cultivation from 95 of the 214 swine samples tested (44.4%). Additionally, five *Salmonella* spp. isolates were retrieved from an equal number of samples (2.3%; Figure 5B).

Seventy-eight isolates (36.5%) presented resistance to 3rd generation cephalosporins and were phenotypically confirmed to produce ESBL (Figure 5C). The number of isolates per ESBL-positive farm ranged from 1 to 9 (median: 2, IQR: 1–3.5), reflecting differences in the number of samples collected per farm according to sow capacity.

Among the ESBL producers identified (Figure 5D–E), *E. coli* predominated with 69 isolates (88.5%) detected in 26 farms (93% of ESBL-positive farms, 76.5% of all farms under study). Other species were detected substantially less frequently: *Proteus mirabilis* (*P. mirabilis*) in four farms (4/78 isolates, 5.1%) and *Klebsiella pneumoniae* (*K. pneumoniae*) in three farms (3/78 isolates, 3.8%), with single isolates of *Salmonella enterica* (*S. enterica*) *IIIb* and *Enterobacter cloacae* (*E. cloacae*) (1.3% each).

Mixed ESBL species were recovered from six farms (21% of ESBL-positive farms; Figure 5F), most commonly *E. coli* with *P. mirabilis* (three farms, 50% of mixed-species farms), suggesting potential cross-contamination or environmental persistence of resistant organisms within these operations. Species diversity per farm ranged from one to three, with maximum diversity (three species) observed in one farm (3% of ESBL-positive farms).

### 3.3. Biosecurity Implementation as a Predictor of ESBL Detection

#### 3.3.1. Biosecurity Scores and ESBL Status

**Figure 6:**
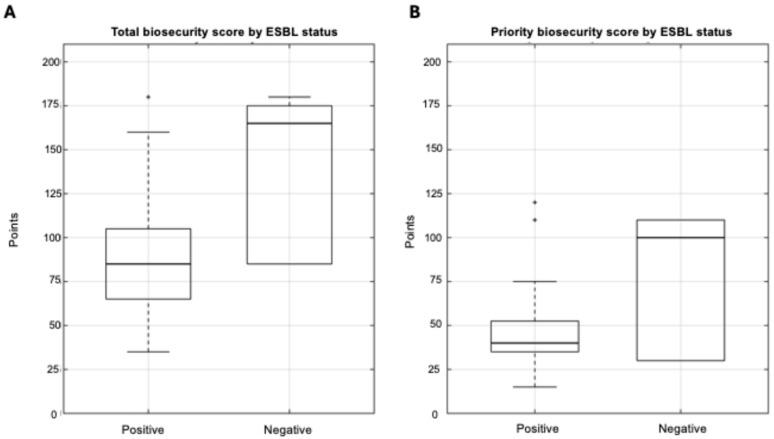
Biosecurity score distributions by ESBL status. Box plots comparing (**A**) total scores and (**B**) priority biosecurity scores between ESBL-positive (*n* = 28) and ESBL-negative farms (*n* = 6). Total biosecurity scores differed significantly between groups (Mann–Whitney U = 28.5, *p* = 0.01), with ESBL-positive farms scoring 48% lower (median: 85 vs. 165 points). Priority scores showed similar patterns but were not statistically significant (*p* = 0.15). Notably, the median total biosecurity score of ESBL-positive farms corresponds exactly to the minimum observed in ESBL-negative farms, suggesting a critical biosecurity implementation threshold below which farms become consistently vulnerable to ESBL contamination.

Analysis of total biosecurity scores revealed significant differences between ESBL-positive and ESBL-negative farms (Figure 6A). For ESBL-positive farms (*n* = 28), scores ranged from 35 to 180 points (median: 85, IQR: 65–105), whereas ESBL-negative farms (*n* = 6) achieved substantially higher scores ranging from 85 to 180 (median: 165, IQR: 85–175). This disparity represented a median difference of 80 points, with ESBL-positive farms scoring approximately 48% lower than ESBL-negative ones (85 vs. 165 points).

Notably, the minimum biosecurity score observed in ESBL-negative farms (85 points) corresponded exactly to the median score of ESBL-positive farms, suggesting a clear threshold effect where farms below this implementation level were consistently associated with ESBL presence. Mann–Whitney U testing confirmed this difference was statistically significant (U = 28.5, *p* = 0.01, r = 0.4), indicating a medium-to-large effect size according to Cohen’s criteria.

Priority biosecurity measures exhibited a similar pattern, although statistical significance was not reached (Figure 6B). ESBL-positive farms achieved scores ranging from 15 to 120 points (median: 40, IQR: 35–53), while ESBL-negative farms scored substantially higher, with a range of 30 to 110 (median: 100, IQR: 30–110). This represented a median difference of 60 points, with ESBL-positive farms implementing approximately 60% fewer priority biosecurity measures compared to ESBL-negative ones. Despite the substantial descriptive differences observed, statistical testing did not reach significance (Mann–Whitney U = 52, *p* = 0.15, r = −0.2).

Point-biserial correlation revealed a significant negative association between ESBL presence and total biosecurity scores (r = −0.51, *p* = 0.01), confirming the strong inverse relationship observed in group comparisons. The association between ESBL presence and priority biosecurity scores, while substantial in magnitude (r = −0.44), did not achieve statistical significance at the conventional 0.05 level (*p* = 0.08).

Spearman rank correlation analysis, which is robust to non-normal distributions of biosecurity parameters, yielded similar patterns. Total biosecurity scores demonstrated a trend toward a strong negative correlation (ρ = −0.49, *p* = 0.09), while priority scores showed a moderate correlation that did not achieve statistical significance (ρ = −0.25, *p* = 0.14). The consistent pattern of weaker associations for priority measures across different analytical approaches suggests that the selected priority parameters may be less discriminatory than the comprehensive biosecurity assessment.

Logistic regression modeling provided additional insights into the predictive relationship between biosecurity implementation and ESBL detection. For total biosecurity scores, each 10-point increase was associated with a 28.8% reduction in the odds of ESBL detection (OR = 0.71, 95% CI: 0.55–0.92, *p* = 0.0097). Model fit statistics supported the predictive utility of total biosecurity scores (Nagelkerke’s R^2^ = 0.37), indicating that over one-third of the variance in ESBL status could be explained by differences in biosecurity implementation.

Priority biosecurity measures demonstrated a similar magnitude of protective effect, with each 10-point increase corresponding to a 29.8 reduction in ESBL presence odds (OR = 0.70, 95% CI: 0.52–0.95). However, this relationship did not achieve statistical significance (*p* = 0.209) and model performance was weaker (Nagelkerke’s R^2^ = 0.26), suggesting that these 14 priority parameters alone may explain no more than one-quarter of the variance in ESBL status.

#### 3.3.2. Species-Specific Biosecurity Associations

To investigate whether biosecurity implementation levels differentially affected specific ESBL-producing species, we examined associations between biosecurity scores and individual pathogen presence. Given the marked predominance of *E. coli* among ESBL producers (88.5% of isolates, detected in 26/34 farms), statistical analyses focused primarily on this species. Additional descriptive comparisons were provided for *P. mirabilis* (*n* = 4 farms) and *K. pneumoniae* (*n* = 3 farms), which occurred with sufficient frequency to identify potential patterns (Figure 5D–E).

#### 3.3.3. *E. coli* ESBL and Biosecurity Implementation

**Figure 7:**
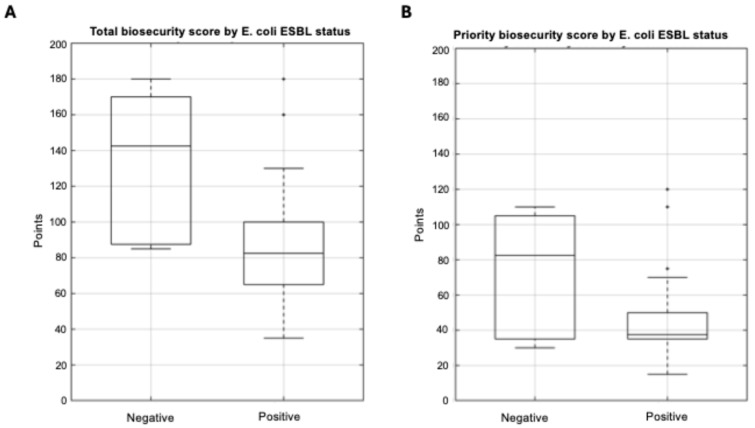
Distribution of biosecurity scores by *E. coli* ESBL status. Boxplots comparing (**A**) total biosecurity score and (**B**) priority biosecurity scores between farms negative (*n* = 8) and positive (*n* = 26) for ESBL-producing *E. coli*. Total biosecurity scores differed significantly between groups (Mann–Whitney U = 36.5, *p* = 0.006), while priority scores showed a non-significant trend (U = 63, *p* = 0.098). The marked difference in total biosecurity scores (median: 142 vs. 82 points) indicates a strong association between comprehensive biosecurity implementation levels and protection against *E. coli* ESBL colonization.

Farms harboring ESBL-producing *E. coli* demonstrated substantially lower biosecurity implementation levels compared to *E. coli*-negative farms. Total biosecurity scores in *E. coli*-positive farms ranged from 35 to 180 points (median: 82, IQR: 65–100), while *E. coli*-negative farms achieved scores of 85 to 180 points (median:142, IQR: 88–170). This difference of 60 points represented a 42% reduction in biosecurity implementation levels (Mann–Whitney U = 36.5, *p* = 0.006, r = 0.47), indicating a large effect size by Cohen’s criteria (Figure 7).

Priority biosecurity measures showed a similar pattern, though with reduced statistical significance. *E. coli*-positive farms ranged from 15 to 120 points (median: 38, IQR: 35–35), while *E. coli*-negative farms achieved scores of 30 to 110 points (median: 82, IQR: 35–105). This difference of 44 points represented a 54% reduction in priority biosecurity implementation levels but did not achieve statistical significance (Mann–Whitney U = 63, *p* = 0.098, r = 0.28).

Logistic regression analysis quantified the protective effect of biosecurity implementation against *E. coli* ESBL colonization. For total biosecurity scores, each 10-point increase was associated with a 25% reduction in the odds of *E. coli* presence (OR = 0.75, 95% CI: 0.59–0.93, *p* = 0.011). The model explained 31% of the variance in *E. coli* status (Nagelkerke’s R^2^ = 0.31).

#### 3.3.4. Biosecurity Patterns for Other ESBL Species

Beyond *E. coli*, which was detected in 26 out of 28 ESBL-positive farms (92.9%), other ESBL-producing species showed distinct biosecurity patterns.

*P. mirabilis* was identified in four farms, with total biosecurity scores ranging from 80 to 160 points (median: 92, IQR: 85–128) and priority scores from 35 to 110 points (median: 45, IQR: 38–80). *K. pneumoniae* ESBL was detected in three farms with total biosecurity scores ranging from 62 to 120 points (median: 80, IQR: 69–110) and priority scores from 35 to 65 points (median: 50, IQR: 39–60), all clustering around or below the study median of 85 points.

The substantial difference between ESBL-negative farms (median: 165) and farms positive for any ESBL species illustrates the protective effect of comprehensive biosecurity implementation. The minimum total biosecurity score observed in ESBL-negative farms (85 points) corresponded closely to the median scores observed in farms with *E. coli* (82 points) and *K. pneumoniae* (80 points), suggesting a potential threshold effect for ESBL colonization risk (Table 3).

## 4. Discussion

This study demonstrates a strong inverse relationship between biosecurity implementation and ESBL prevalence in Greek swine farms, with comprehensive biosecurity measures providing greater protection than priority measures alone. The 82% farm-level ESBL prevalence observed substantially exceeds rates reported in other European countries [8,23], highlighting an urgent need for intervention in the Greek swine sector. Our findings highlight that improved biosecurity measures lead to reduced phenomena of ESBL-producing bacteria presence. These results are in accordance with previous studies in which inadequate biosecurity measures correlated with higher AMU [25,30,31].

Perhaps the most intriguing finding was the absence of significant associations between farm size and individual biosecurity parameters, despite strong overall correlations. This paradox reveals that biosecurity is fundamentally a systems-level phenomenon, where cumulative marginal improvements across multiple measures create synergistic protective effects that cannot be achieved through isolated interventions. This paradoxical finding—where supposedly “priority” measures show greater variability and weaker associations with ESBL prevention—suggests a need to reassess which biosecurity components truly provide the most protection under field conditions. Moreover, this result is in contrast with the study that our biosecurity assessment system was based on [28]. Previous research examined the influence of basic biosecurity measures, such as all-in–all-out systems, single animal sources, etc., in the presence of AMR [17,26,32]. In this study, parameters that were considered secondary or absent in previous research [17], such as written biosecurity guidelines for visitors at the entrance or the use of gloves in the handling of dead animals, were examined and ultimately shown to contribute to the reduction of ESBL-producing *Enterobacteriaceae* presence. Our finding has profound implications for biosecurity strategy. Rather than focusing resources on perfecting specific “critical control points”, farms appear to benefit more from distributed improvements across all biosecurity domains. Each individual improvement may be statistically insignificant, yet their combined effect produces substantial protection against ESBL colonization. This challenges the prevailing reductionist approach to biosecurity and suggests that holistic, multi-faceted strategies may be more effective. This systems-level effect represents a potential paradigm shift in understanding biosecurity effectiveness. Rather than focusing resources on perfecting specific ‘critical control points’, our findings suggest that farms benefit more from distributed improvements across all biosecurity domains. This may help explain why previous studies examining individual biosecurity measures have shown inconsistent results for AMR prevention—they were searching for individual effects that only manifest at the systems level. The practical implication is clear: achieving 5-point improvements across 20 measures likely provides better ESBL protection than perfect implementation of 8–10 ‘priority’ measures. The observation that the minimum biosecurity score in ESBL-negative farms (85 points) corresponded exactly to the median score of ESBL-positive farms suggests a critical implementation threshold. Farms scoring below 85 points (35% of maximum) were consistently vulnerable to ESBL contamination. This threshold effect has important implications for establishing minimum biosecurity standards in swine production. The median implementation of only 35% of recommended biosecurity measures indicates substantial room for improvement across the Greek swine industry. The even lower implementation of priority measures (29% median) suggests that fundamental pathogen prevention guidelines face greater adoption barriers than supplementary practices. This pattern may reflect higher technical complexity, resource requirements or practical constraints associated with core biosecurity measures. Our study’s results are alarming regarding the biosecurity level in Greek pig farms. Findings from other countries worldwide reported higher biosecurity scores, highlighting the urgent need for more professionalism in that sector [33,34].

The strong positive association between farm size and biosecurity implementation likely reflects differential access to technical expertise and financial resources. Farms with >800 sows typically have greater resources to invest in infrastructure, training and systematic biosecurity protocols, and these results are also in agreement with previous studies [12,34]. However, our findings suggest that even farms with <200 sows can achieve protection through distributed improvements across multiple biosecurity domains rather than requiring excellence in specific high-cost interventions. The heterogeneity in farm sizes and biosecurity scores within our sample reflects the diverse nature of Greek swine production. This diversity presents both challenges and opportunities for targeted interventions based on farm scale and current biosecurity status.

*E. coli* dominated the ESBL landscape (88.5% of isolates), consistent with global patterns in swine production [35]. The stronger association between biosecurity and *E. coli* compared to other species may reflect this organism’s transmission characteristics and environmental persistence. The presence of *P. mirabilis* and *K. pneumoniae* at intermediate biosecurity levels suggests species-specific vulnerability patterns that warrant further investigation. The occurrence of mixed ESBL species in 21% of positive farms indicates potential cross-contamination or shared resistance selection pressures within operations. This finding emphasizes the need for comprehensive approaches targeting multiple bacterial species simultaneously.

The convergence of human, animal and ecosystem health—conceptualized within the One Health framework—is increasingly acknowledged as a critical determinant in the emergence and dissemination of AMR. This interface constitutes not only a driver of resistance evolution but also an underutilized opportunity for comprehensive AMR surveillance [36]. Indeed, AMR exemplifies a quintessential One Health challenge [37,38]. High rates of farms positive for ESBL-producing *Enterobacteriaceae* continue to be reported in swine, exceeding those in other livestock such as ruminants, which also exhibit resistance to antimicrobials critical for human health, such as colistin, ultimately jeopardizing human health [39,40]. Antimicrobial resistance genes (ARGs) epitomize the interconnectedness of humans, animals, plants and their shared environments. The inappropriate use of antimicrobials fosters conditions conducive to the selection and stabilization of resistance genes, which may subsequently disseminate to humans and animals through contaminated food products or environmental reservoirs. Beyond the human–animal interface, the environment assumes a dual role, both as a conduit for dissemination and as a reservoir for the persistence of AMR. This underscores the necessity of surveillance strategies explicitly aligned with the One Health paradigm [41]. Exacerbating factors include limited access to veterinary services, inadequate knowledge regarding antimicrobial therapies, and the availability of antimicrobials without prescription, all of which contribute to misuse and subsequent environmental contamination. Addressing these challenges requires the establishment of educational outreach platforms and the implementation of evidence-based public health policies that promote judicious AMU within the swine industry [36]. Regular on-farm training—preferably facilitated by experts on an annual basis—represents a pragmatic avenue for advancing antimicrobial stewardship. When integrated with training in hygiene and biosecurity procedures, such interventions are expected to yield systemic improvements, particularly within professional zones and their transitional areas, which constitute the principal targets for cleaning and disinfection measures [42]. Several authors have suggested that non-compliance with biosecurity measures is frequently associated with insufficient training of farm personnel and ineffective communication between farm workers and technical service providers [42,43,44]. This is particularly evident in relation to the understanding of how individual measures contribute to the prevention of disease transmission. To address this gap and strengthen communication between farmers and biosecurity advisors, Scollo et al. recommend the implementation of a tailored biosecurity plan [43].

Several limitations should be considered when interpreting these findings. First, the cross-sectional design prevents definitive causal inference regarding the biosecurity–ESBL relationship. Second, the relatively small number of ESBL-negative farms may limit statistical power for some comparisons, though the large effect sizes observed suggest robust associations. Third, our biosecurity assessment relied on a categorical scoring system developed for this study. While this three-tier system (0/5/10) demonstrated strong discriminatory power and significant associations with ESBL prevalence, we acknowledge that more granular scoring might reveal additional patterns, particularly for farms in the middle range of the implementation. This categorical approach was chosen to reflect how biosecurity measures exist in practice and to enhance reproducibility, but future research using continuous or more detailed scoring systems could potentially identify subtle relationships not captured here. Finally, sampling was limited to piglets, potentially underestimating overall farm ESBL prevalence, as adult animals may harbor different resistance patterns. Future research should employ longitudinal designs to establish temporal relationships between biosecurity implementation and ESBL emergence. Investigation of specific biosecurity measure combinations that provide synergistic protection could inform more efficient intervention strategies. Additionally, economic analyses comparing the costs of comprehensive biosecurity implementation versus losses from ESBL-related treatment failures would provide valuable decision-making support for producers.

## 5. Conclusions

This study provides strong evidence that comprehensive biosecurity implementation serves as a critical barrier against antimicrobial-resistant bacteria in swine operations. The absence of significant individual parameter associations despite strong aggregate effects reveals biosecurity as a systems-level phenomenon, challenging conventional approaches that focus on critical control points. These findings argue for a paradigm shift from focusing on individual “critical control points” to promoting distributed improvements across multiple biosecurity domains. With 82% of Greek swine farms harboring ESBL-producing bacteria and median biosecurity implementation at only 35% of recommended levels, there is both urgent need and substantial opportunity for improvement in the Greek swine sector.

## Figures and Tables

**Table 1:** Priority biosecurity parameters and scoring criteria (*n* = 14, maximum 140 points).

Parameter	Scoring Criteria	Points
Presence of a sign to declare prohibited access to the farm	Absence/Presence	0/10
Unique entrance in the farm	Multiple entrances/Unique access	0/10
Stable and perimetral fence covering the territory of the farm	Absence of perimetral fence/Disabled fence/Presence of perimetral fence	0/5/10
Presence of bird-proof nets in the barns	Absence/Presence	0/10
External feed loading	Absence/Presence	0/10
Shower for all visitors at the entrance	Absence/Presence	0/10
Presence of dressing room for visitors	Absence/Presence	0/10
Clean and dirty zones at visitors and personnel dressing room	Absence/Presence	0/10
Application of disinfection for all vehicles	No implementation/Implementation	0/10
Presence of visitors parking	Absence/Presence	0/10
Prohibited access for trucks inside the farm	No implementation/Implementation	0/10
Equipment for visitors provided by the farm	Absence/Presence	0/10
Equipment for personnel provided by the farm	Absence/Presence	0/10
Truck cleaning at farm’s entrance	No implementation/Implementation	0/10

**Table 2:** Supplementary biosecurity parameters and scoring criteria (*n* = 21, maximum 105 points).

Parameter	Scoring Criteria	Points
Pig breeding by neighbors	Breeding/Lack of neighbors breeding pigs	0/5
Cover of land around the farm	Cover/Absence of any activity	0/5
Biosecurity protocol for visitors	Absence/Presence	0/5
Visitors entrance	Frequent/Rare	0/5
Entrance of trucks transporting living animals	Entry/Restricted access	0/5
Vehicles dedicated to specific production site	No specific trucks/Specific truck for each farm’s site	0/5
No truck driver access in the farm	Access/No access	0/5
Presence of disinfection equipment room	Absence/Presence	0/5
Chemical water treatment	No application/Application	0/5
Water chemical examination	No application/Application	0/5
Equipment disinfection during entrance	No application/Application	0/5
Other animal species breeding in the farm	Presence/Absence	0/5
Restricted staff contact with other farm animals	Contact/No contact	0/5
Unique source for animal replacement	Multiple sources/Unique sources	0/5
All in–all out system	Absence/Presence	0/5
Special equipment per pen	Absence/Presence	0/5
Maintenance of sick animal pen	Absence/Presence	0/5
Application of rodent control	No implementation/Implementation	0/5
Application of flies control	No implementation/Implementation	0/5
Implementation of biosecurity training for farm personnel	No implementation/Implementation	0/5
Use of disposable gloves to handle dead animals	No use/Use	0/5

**Table 3:** Distribution of biosecurity scores by ESBL species presence across the 34 participating farms. Categories are not mutually exclusive as some farms harbored multiple ESBL species. The number of farms represents those positive for each species out of 28 ESBL-positive farms, except for the “No ESBL” category, which represents 6 of the 34 total farms studied.

ESBL Species	Farms	Total Biosecurity Score		Priority Biosecurity Score	
	No	Median (IQR)	Range	Median (IQR)	Range
*E. coli*	26	82 (65–100)	35–180	38 (35–50)	15–120
*P. mirabilis*	4	92 (85–128)	80–160	45 (38–80)	35–110
*K. pneumoniae*	3	80 (69–110)	62–110	50 (39–60)	35–65
No ESBL	6	165 (85–175)	85–110	100 (30–110)	30–110

## Data Availability

The original contributions presented in this study are included in the article/Supplementary Material. Further inquiries can be directed to the corresponding author.

## Supplementary Materials

The following supporting information can be downloaded at https://www.mdpi.com/article/10.3390/life15101629/s1, File S1: Questionnaire; File S2: Antimicrobial Resistance in ESBL isolates.

## Abbreviations

The following abbreviations are used in this manuscript:

ESBLs	Extended-spectrum β-lactamases
WHO	World Health Organization
AMR	Antimicrobial resistance
ASF	African Swine Fever
PRRSV	Porcine Reproductive and Respiratory Syndrome Virus
AMU	Antimicrobial use
EU	European Union
FDR	False discovery rate
ARGs	Antimicrobial resistance genes

## References

[1] Bud R. (2007). Antibiotics: The epitome of a wonder drug. BMJ.

[2] Gwynn M.N., Portnoy A., Rittenhouse S.F., Payne D.J. (2010). Challenges of antibacterial discovery revisited. Ann. N. Y. Acad. Sci..

[3] Center for Veterinary Medicine Summary Report on Antimicrobials Sold or Distributed for Use in Food-Producing Animals. US Food and Drug Administration. https://www.fda.gov/media/163739/download?attachment.

[4] Maslikowska J.A., Walker S.A.N., Elligsen M., Mittmann N., Palmay L., Daneman N., Simor A. (2016). Impact of infection with extended-spectrum β-lactamase-producing *Escherichia coli* or *Klebsiella* species on outcome and hospitalization costs. J. Hosp. Infect..

[5] Tseng C.H., Liu C.W., Liu P.Y. (2023). Extended-Spectrum β-Lactamases (ESBL) Producing Bacteria in Animals. Antibiotics.

[6] Filioussis G., Kachrimanidou M., Christodoulopoulos G., Kyritsi M., Hadjichristodoulou C., Adamopoulou M., Tzivara A., Kritas S.K., Grinberg A. (2020). Short communication: Bovine mastitis caused by a multidrug-resistant, mcr-1-positive (colistin-resistant), extended-spectrum β-lactamase-producing *Escherichia coli* clone on a Greek dairy farm. J. Dairy. Sci..

[7] Athanasakopoulou Z., Reinicke M., Diezel C., Sofia M., Chatzopoulos D.C., Braun S.D., Reissig A., Spyrou V., Monecke S., Ehricht R. (2021). Antimicrobial Resistance Genes in ESBL-Producing *Escherichia coli* Isolates from Animals in Greece. Antibiotics.

[8] Tsekouras N., Athanasakopoulou Z., Diezel C., Kostoulas P., Braun S.D., Sofia M., Monecke S., Ehricht R., Chatzopoulos D.C., Gary D. (2022). Cross-Sectional Survey of Antibiotic Resistance in Extended Spectrum β-Lactamase-Producing *Enterobacteriaceae* Isolated from Pigs in Greece. Animals.

[9] Xi M., Wu Q., Wang X., Yang B., Xia X., Li D. (2015). Characterization of extended-spectrum beta-lactamase-producing *Escherichia coli* strains isolated from retail foods in Shaanxi Province, China. J. Food Prot..

[10] Yu T., Jiang X., Fu K., Liu B., Xu D., Ji S., Zhou L. (2013). Detection of extended-spectrum b-lactamase and plasmid-mediated quinolone resistance determinants in *Escherichia coli* isolates from retail meat in China. J. Food Prot..

[11] Bergšpica I., Kaprou G., Alexa E.A., Prieto M., Alvarez-Ordóñez A. (2020). Extended Spectrum β-Lactamase (ESBL) Producing *Escherichia coli* in Pigs and Pork Meat in the European Union. Antibiotics.

[12] Laanen M., Persoons D., Ribbens S., de Jong E., Callens B., Strubbe M., Maes D., Dewulf J. (2013). Relationship between biosecurity and production/antimicrobial treatment characteristics in pig herds. Vet. J..

[13] Sahlström L., Virtanen T., Kyyrö J., Lyytikäinen T. (2014). Biosecurity on Finnish cattle, pig and sheep farms—Results from a questionnaire. Prev. Vet. Med..

[14] Papatsiros V.G. (2013). Biosecurity management practices for the prevention and control of PRRS. Porc. Res..

[15] Alarcón L.V., Allepuz A., Mateu E. (2021). Biosecurity in pig farms: A review. Porc. Health Manag..

[16] Otake S., Yoshida M., Dee S.A. (2024). Review of Swine Breeding Herd Biosecurity in the United States to Prevent Virus Entry Using Porcine Reproductive and Respiratory Syndrome Virus as a Model Pathogen. Animals.

[17] Stygar A.H., Chantziaras I., Toppari I., Maes D., Niemi J.K. (2020). High biosecurity and welfare standards in fattening pig farms are associated with reduced antimicrobial use. Animal.

[18] Postma M., Backhans A., Collineau L., Loesken S., Sjölund M., Belloc C., Emanuelson U., Grosse Beilage E., Stärk K.D.C., Dewulf J. (2016). MINAPIG consortium. The biosecurity status and its associations with production and management characteristics in farrow-to-finish pig herds. Animal.

[19] Mallioris P., Teunis G., Lagerweij G., Joosten P., Dewulf J., Wagenaar J.A., Stegeman A., Mughini-Gras L. (2022). Biosecurity, and antimicrobial use in broiler farms across nine European countries: Towards identifying farm-specific options for reducing antimicrobial usage. Epidemiol. Infect..

[20] Robertson I.D. (2020). Disease Control, Prevention and On-Farm Biosecurity: The Role of Veterinary Epidemiology. Engineering.

[21] Postma M., Vanderhaeghen W., Sarrazin S., Maes D., Dewulf J. (2017). Reducing antimicrobial usage in pig production without jeopardizing production parameters. Zoonoses Public Health.

[22] Chantziaras I., Boyen F., Callens B., Dewulf J. (2013). Correlation between veterinary antimicrobial use and antimicrobial resistance in food-producing animals: A report on seven countries. J. Antimicrob. Chem..

[23] Dohmen W., Liakopoulos A., Bonten M.J.M., Mevius D.J., Heederik D.J.J. (2023). Longitudinal Study of Dynamic Epidemiology of Extended-Spectrum Beta-Lactamase-Producing *Escherichia coli* in Pigs and Humans Living and/or Working on Pig Farms. Microbiol. Spectr..

[24] Fischer J., Hille K., Ruddat I., Mellmann A., Köck R., Kreienbrock L. (2017). Simultaneous occurrence of MRSA and ESBL-producing *Enterobacteriaceae* on pig farms and in nasal and stool samples from farmers. Vet. Microbiol..

[25] Raasch S., Postma M., Dewulf J., Stärk K.D.C., Grosse Beilage E. (2018). Association between antimicrobial usage, biosecurity measures as well as farm performance in German farrow-to-finish farms. Porc. Health Manag..

[26] Caekebeke N., Jonquiere F.J., Ringenier M., Tobias T.J., Postma M., van den Hoogen A., Houben M.A.M., Velkers F.C., Sleeckx N., Stegeman J.A. (2020). Comparing Farm Biosecurity and Antimicrobial Use in High-Antimicrobial-Consuming Broiler and Pig Farms in the Belgian-Dutch Border Region. Front. Vet. Sci..

[27] National Academies Press (2012). Nutrient Requirements of Swine.

[28] Silva G.S., Leotti V.B., Castro S.M.J., Medeiros A.A.R., Silva A.P.S.P., Linhares D.C.L., Corbellini L.G. (2019). Assessment of biosecurity practices and development of a scoring system in swine farms using item response theory. Prev. Vet. Med..

[29] Microbiology of the Food Chain—Horizontal Method for the Detection, Enumeration and Serotyping of Salmonella—Part 1: Detection of Salmonella spp.

[30] Dhaka P., Chantziaras I., Vijay D., Bedi J.S., Makovska I., Biebaut E., Dewulf J. (2023). Can Improved Farm Biosecurity Reduce the Need for Antimicrobials in Food Animals? A Scoping Review. Antibiotics.

[31] Jaleta M., Junker V., Kolte B., Börger M., Werner D., Dolsdorf C., Schwenker J., Hölzel C., Zentek J., Amon T. (2024). Improvements of weaned pigs barn hygiene to reduce the spread of antimicrobial resistance. Front. Microbiol..

[32] Salgado-Caxito M., Léon D., Bardales O., Jara L.M., Medrano P., Murga C., Pérez V., Aylas-Jurado B., Su-Tello R., Najarro J. (2025). Unexplained High Prevalence of ESBL-*Escherichia coli* Among Cattle and Pigs in Peru. Antibiotics.

[33] Wang J., Shi Z., Hu X. (2023). Status, evaluation, and influencing factors of biosecurity levels in pig farms in China. BMC Vet. Res..

[34] Zhang W., Lu Q. (2024). The impact of epidemic experiences on biosecurity behavior of pig farmers: An analysis based on protection motivation theory. One Health.

[35] Mandujano-Hernández A., Martínez-Vázquez A.V., Paz-González A.D., Herrera-Mayorga V., Sánchez-Sánchez M., Lara-Ramírez E.E., Vázquez K., de Jesús de Luna-Santillana E., Bocanegra-García V., Rivera G. (2024). The Global Rise of ESBL-Producing *Escherichia coli* in the Livestock Sector: A Five-Year Overview. Animals.

[36] da Silva S.K.S.M., Fuentes-Castillo D.A., Ewbank A.C., Sacristán C., Catão-Dias J.L., Sevá A.P., Lincopan N., Deem S.L., Feitosa L.C.S., Catenacci L.S. (2024). ESBL-Producing *Enterobacterales* at the Human–Domestic Animal–Wildlife Interface: A One Health Approach to Antimicrobial Resistance in Piauí, Northeastern Brazil. Vet. Sci..

[37] Robinson T.P., Bu D.P., Carrique-Mas J., Fèvre E.M., Gilbert M., Grace D., Hay S.I., Jiwakanon J., Kakkar M., Kariuki S. (2016). Antibiotic Resistance Is the Quintessential One Health Issue. Trans. R. Soc. Trop. Med. Hyg..

[38] Ahmed N.A., Gulhan T. (2024). Determination of Antibiotic Resistance Patterns and Genotypes of *Escherichia coli* Isolated from Wild Birds. Microbiome.

[39] Kim J.-I., Moon B.-Y., Ali M.S., Kang H.-S., Choi J.-H., Kim J.-M., Park S.-C., Lim S.-K. (2025). High prevalence of *bla*_CTX-M-55_-carrying *Escherichia coli* in both ceftiofur-use and non-use pig farms. Appl. Environ. Microbiol..

[40] Ventura N.K.O., Freitas L.R., Sousa F.A., Cossi M.V.C., Nero L.A., Yamatogi R.S. (2025). Colistin and β-lactam resistance in *Escherichia coli* isolates from bovines, swine, and humans. J. Infect. Dev. Ctries..

[41] McEwen S.A., Collignon P.J. (2018). Antimicrobial Resistance: A One Health Perspective. Microbiol. Spectr..

[42] Scollo A., Perrucci A., Stella M.C., Ferrari P., Robino P., Nebbia P. (2023). Biosecurity and Hygiene Procedures in Pig Farms: Effects of a Tailor-Made Approach as Monitored by Environmental Samples. Animals.

[43] Scollo A., Levallois P., Fourichon C., Motta A., Mannelli A., Lombardo F., Ferrari P. (2022). Monitoring Means and Results of Biosecurity in Pig Fattening Farms: Systematic Assessment of Measures in Place and Exploration of Biomarkers of Interest. Animals.

[44] Casal J., De Manuel A., Mateu E., Martín M. (2007). Biosecurity measures on swine farms in Spain: Perceptions by farmers and their relationship to current on-farm measures. Prev. Vet. Med..

