# Supplementary material for: Can Improved Biosecurity Measures Reduce the Presence of the Most Common ESBL-Producing Enterobacteriaceae? A Study from Greek Pig Farms
Source: Life (Basel). 2025 Oct 19;15(10):1629. doi: 10.3390/life15101629 (PMC12565144; doi:10.3390/life15101629)
Supplement: Supplementary file 1 [file life-15-01629-s001.zip › S1. Questionnaire.pdf]

Questionnaire for Biosecurity Level Assessment

Respondent Information

1. **Farmer's Name:**

2. **Farm Name:**

3. **Date of Assessment:**

4. **Veterinarian/Assessor Name:**

Biosecurity Level Assessment Parameters

First Category: **High Importance Parameters**
For each parameter, please indicate the score based on the descriptions provided (0=absence, 5=disabled, 10=presence).

1. **Sign to declare prohibited access to the farm:**

[ ] 0 (absence)
[ ] 10 (presence)

2. **Unique entrance to the farm:**

[ ] 0 (absence)
[ ] 10 (presence)

3. **Stable and perimetral fence covering the territory of the farm:**

[ ] 0 (absence)
[ ] 5 (disabled)
[ ] 10 (presence)

4. **Presence of bird-proof nets in barns:**

[ ] 0 (absence)
[ ] 10 (presence)

5. **External feed loading protocol:**

[ ] 0 (implementation)
[ ] 10 (no implementation)

6. **Shower facility for all visitors at the entrance:**
[ ] 0 (absence)
[ ] 10 (presence)

7. **Presence of dressing room for visitors:**

[ ] 0 (absence)
[ ] 10 (presence)

8. **Clean and dirty zones in visitors' and personnel dressing rooms:**

[ ] 0 (absence)
[ ] 10 (presence)

9. **Application of disinfection for all vehicles:**

[ ] 0 (implementation)
[ ] 10 (no implementation)

10. **Presence of visitors parking area:**

[ ] 0 (absence)
[ ] 10 (presence)

11. **Prohibited access for trucks inside the farm:**

[ ] 0 (implementation)
[ ] 10 (no implementation)

12. **Equipment for visitors provided by the farm:**

[ ] 0 (implementation)
[ ] 10 (no implementation)

13. **Equipment for personnel provided by the farm:**

[ ] 0 (implementation)
[ ] 10 (no implementation)

14. **Truck cleaning procedures at farm's entrance:**

[ ] 0 (implementation)
[ ] 10 (no implementation)

Second Category: **Lower Importance Parameters**
For each parameter, please indicate the score based on the descriptions provided (0=absence, 5=presence).

15. **Pigs' breeding by neighbors:**

[ ] 0 (breeding)
[ ] 5 (no breeding)

16. **Cover of land around the farm:**

[ ] 0 (covered)
[ ] 5 (no cover)

17. **Biosecurity protocol for visitors:**

[ ] 0 (no implementation)
[ ] 5 (implementation)

18. **Frequency of visitors' entrance:**

[ ] 0 (frequent)
[ ] 5 (rare)

19. **Entrance of trucks transporting live animals:**

[ ] 0 (entry)
[ ] 5 (restricted access)

20. **Vehicles dedicated to specific production site:**

[ ] 0 (no specific trucks)
[ ] 5 (specific truck for each site)

21. **No truck-drivers access to the farm:**

[ ] 0 (access)
[ ] 5 (no access)

22. **Presence of disinfection equipment room:**

[ ] 0 (absence)
[ ] 5 (presence)

23. **Chemical water treatment application:**
[ ] 0 (no application)
[ ] 5 (application)

24. **Water chemical examination conducted:**

[ ] 0 (no application)
[ ] 5 (application)

25. **Equipment disinfection during entrance:**
[ ] 0 (no application)
[ ] 5 (application)

26. **Other animal species breeding within the farm:**
[ ] 0 (presence)
[ ] 5 (absence)

27. **Restricted staff's contact with other farm animals:**
[ ] 0 (contact)
[ ] 5 (no contact)

28. **Unique source for animals' replacement:**
[ ] 0 (multiple sources)
[ ] 5 (unique source)

29. **All in-all out system in place:**
[ ] 0 (absence)
[ ] 5 (presence)

30. **Special equipment per pen:**
[ ] 0 (absence)
[ ] 5 (presence)

31. **Maintenance of sick animals' pen:**
[ ] 0 (absence)
[ ] 5 (presence)

32. **Implementation of rodent control measures:**
[ ] 0 (no implementation)
[ ] 5 (implementation)

33. **Implementation of flies control measures:**
[ ] 0 (no implementation)
[ ] 5 (implementation)

34. **Performance of biosecurity training for farm personnel:**
[ ] 0 (no implementation)
[ ] 5 (implementation)

35. **Use of disposable gloves to handle dead animals:**
[ ] 0 (no use)
[ ] 5 (use)

Total Score from Category 1 (High Importance): ____ / 140

Total Score from Category 2 (Lower Importance): ____ / 105

Overall Score: ____ / 245

Additional Comments:
Please provide any additional comments or observations regarding biosecurity measures at the farm:

____

____
